# Synthesis and *In Vitro* Antiproliferative Activity of Novel Androst-5-ene Triazolyl and Tetrazolyl Derivatives

**DOI:** 10.3390/molecules16064786

**Published:** 2011-06-09

**Authors:** Zalán Kádár, Dóra Kovács, Éva Frank, Gyula Schneider, Judit Huber, István Zupkó, Tibor Bartók, János Wölfling

**Affiliations:** 1 Department of Organic Chemistry, University of Szeged, Dóm tér 8, H-6720 Szeged, Hungary; 2 Department of Pharmacodynamics and Biopharmacy, University of Szeged, Eötvös u. 6, H-6720 Szeged, Hungary; 3 Faculty of Engineering, University of Szeged, Moszkvai krt. 5-7, H-6725 Szeged, Hungary; 4 Fumizol Ltd., Moszkvai krt. 5-7, H-6725 Szeged, Hungary

**Keywords:** click chemistry, steroid azides, triazoles, tetrazoles, CuAAC

## Abstract

A straightforward and reliable method for the regioselective synthesis of steroidal 1,4-disubstituted triazoles and 1,5-disubstituted tetrazoles via copper(I)-catalyzed cycloadditions is reported. Heterocycle moieties were efficiently introduced onto the starting azide compound 3β-acetoxy-16β-azidomethylandrost-5-en-17β-ol through use of the “click” chemistry approach. The antiproliferative activities of the newly-synthesized triazoles were determined *in vitro* on three human gynecological cell lines (HeLa, MCF7 and A2780) using the microculture tetrazolium assay.

## 1. Introduction

In the past few years, the Huisgen 1,3-dipolar cycloaddition of azides and terminal alkynes to form triazoles has received revived attention. Since the independent reports of Sharpless [1] and Meldal [2], this process has become the most extensively studied “click” reaction, as evidenced by a nearly exponential growth in the number of related publications. Compared with the non-catalyzed version the copper(I)-catalyzed azide-alkyne cycloaddition (CuAAC) has certain advantageous properties, such as regioselectivity, versatility, high conversions and the lack of by-products [3,4,5]. Moreover, this process performs well in most common laboratory solvents and usually does not require protection from oxygen and water, making it an ideal tool for the synthesis of libraries for initial screening and structure-activity profiling.

In contrast, other 1,3-dipolar cycloadditions between nitriles and organic azides to afford tetrazoles generally requires highly electrophilic nitrile carbon atoms and harsh conditions [6]. Demko and Sharpless recently reported the syntheses of some 1,5-disubstituted tetrazoles [7,8] under solvent-free conditions at 100-120 °C. Furthermore, a series of potential catalysts for these reactions were investigated by Vilarrasa *et al*. [9], with the aim of achieving milder conditions. The commercially available or easily prepared [10] copper(I) triflate was observed to be the most efficient catalyst.

To the best of our knowledge, only a few examples are to be found in the literature in which 1,3-dipolar cycloadditions have been applied to steroidal azides [11,12,13,14,15]. Thus, in continuation of our program on the synthesis of steroidal heterocycles [16,17,18,19], we set out to develop an effective route for the production of novel steroidal triazoles and tetrazoles through use of the “click” chemistry approach. The present paper reports the syntheses of D-ring-substituted androst-5-ene derivatives containing a 1,4-disubstituted triazole (compounds **6a**-**j**, **7a**-**j**) or a 1,5-disubstituted tetrazole moieties (compounds **9a**-**e**, **11a**-**e**).

Five-membered nitrogen heterocycles play an important role in biological systems. Not surprisingly, a number of compounds containing 1,2,3-triazoles are found to exhibit a broad spectrum of biological activities, including antimicrobial [20], anti-HIV [21], antiallergic [22] and antiviral [23] effects. A set of 1,2,3-triazol-1-yl podophyllotoxin derivatives were synthesized and some of them proved to be more potent in inhibiting the growth of human cancer cells than etoposide [24]. Several benzotriazoles proved to be novel and potent antiproliferative agents and some of them exhibited nanomolar IC_50_ values against human adherent cancer cell lines [25]. A series of substituted tetrazol-5-ones have been synthesized and three of them were found to inhibit leukemia and breast cancer growth *in vitro* [26]. On the basis of these reports, the triazole and tetrazole ring systems can therefore be regarded as structural blocks suitable for improvement of the anticancer properties of potential pharmacons. Since we reported a set of androstene-fused arylpyrazolines as antiproliferative compounds, it appeared rational to improve the pharmacological profile of the skeleton by means of the introduction of a triazole or tetrazole moiety [19]. Moreover, some 21-triazolyl derivatives of pregnenolone were recently reported as potential anticancer agents by Banday *et al*. [15]. Thus, the newly-prepared triazolyl derivatives were screened *in vitro* for their activities against a panel of three human malignant cell lines.

## 2. Results and Discussion

### 2.1. Synthesis

**Scheme 1:**
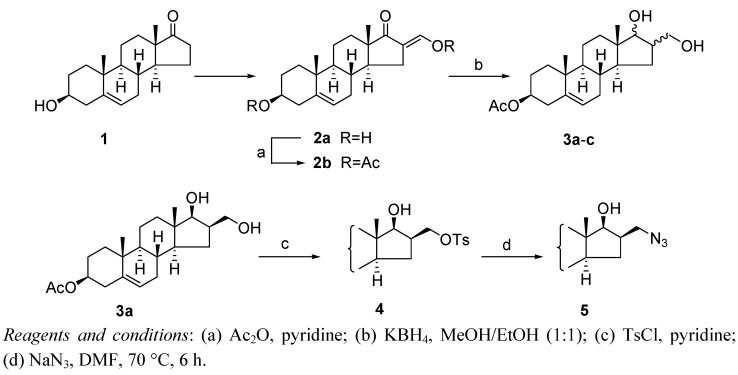
Synthesis of the steroid azide.

To prepare novel steroid triazoles via 1,3-dipolar cycloaddition, 3β-acetoxy-16β-azidomethylandrost-5-en-17β-ol (**5**) was chosen as starting compound. The synthetic strategy for the preparation of the starting azide is illustrated in Scheme 1.

The reaction of 3β-hydroxy-16-hydroxymethylideneandrost-5-en-17-one (**2a**) [27] with acetic anhydride in pyridine medium afforded the diacetate **2b** in excellent yield. According to our earlier observation [28], the reduction of 3β-acetoxy-16-acetoxymethylideneandrost-5-en-17-one (**2b**) with KBH_4_ under pH-controlled conditions leads to three diol isomers. Two of them (compounds **3a**, **3b**), containing 17β-hydroxy groups with opposite configurations at C-16, were isolated in nearly identical amounts, while the third one, the 16β,17α isomer **3c**, was obtained in a significantly smaller quantity (~5%). After separation of the 16β,17β-hydroxymethyl isomer **3a** by flash chromatography, the primary hydroxy group in **3a** was converted into a good leaving group with *p*-toluenesulfonyl chloride. Finally, the crude product **4** was used without purification for further nucleophilic substitution with NaN_3_ in DMF to provide the desired 3β-acetoxy-16β-azidomethylandrost-5-en-17β-ol (**5**) in good yield.

Several D-ring-substituted androst-5-ene derivatives containing a 1,2,3-triazole ring (compounds **6a**-**j**) were synthesized by the reaction of **5** with various terminal alkynes through use of the “click” chemistry approach (Table 1). Although there are a number of methods for generation of the active catalyst [29], one of the most common techniques was chosen. Thus, the Cu(I) species was generated *in situ* by the reduction of CuSO_4_·5H_2_O with sodium ascorbate to minimize the formation of by-products. Furthermore, a mixture of CH_2_Cl_2_ as solvent and water as co-solvent was employed to eliminate the need for ligands and to simplify the reaction protocol [30].

In all cases, total consumption of the starting compound was observed within 1-4 h at room temperature. The reactions were very selective, and triazole products could be isolated in 78-93% yields. The trace quantities of copper and reagents remaining in the reaction mixtures were removed by flash chromatography. Treatment of **6a**-**j** containing a 3β-acetyl group with KOH in MeOH at 50 °C resulted in the corresponding 3β-hydroxy compounds **7a**-**j** in good yields (Table 1).

**Table 1:** Synthesis of the 1,4-disubstituted steroidal triazoles and hydrolysis of their 3-acetyl groups. 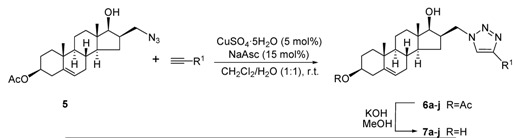

Entry	R1	Triazoles (6 and 7)	Yield ^a^ (%) of 6	Yield ^a^ (%) of 7
1	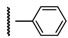	**a**	89	82
2	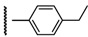	**b**	91	81
3	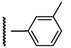	**c**	91	88
4	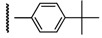	**d**	93	85
5	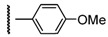	**e**	86	86
6	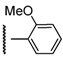	**f**	83	87
7	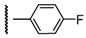	**g**	85	91
8	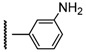	**h**	90	82
9	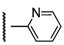	**i**	87	90
10	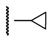	**j**	78	83

^a^ Yields of purified isolated products.

These outstanding results encouraged us to investigate another example of “click” reactions. The intermolecular [3+2] cycloadditions between the steroid azides **5** and **10** and several nitriles **8a**-**e** containing an electron-withdrawing group (EWG) afforded the desired 1,5-disubstituted steroidal tetrazoles **9a**-**e** and **11a**-**e**. As mentioned earlier, highly electrophilic nitrile carbon atoms are required for successful addition [9]; some commercially available acyl cyanides and cyanoformates were therefore chosen as reagents. In all cases, the reactions were carried out at room temperature, with stirring for 2 days, 10 mol % copper(I) complex Cu_2_(OTf)_2_·C_6_H_6_ (OTf = O_3_SCF_3_) being used as catalyst. The newly-synthesized tetrazolyl compounds could be isolated in 45-72% yields after purification by column chromatography (Table 2).

**Table 2:** Synthesis of the 1,5-disubstituted steroidal tetrazoles. 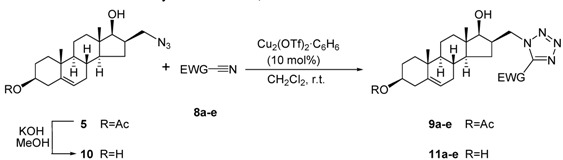

Entry	Reactant	EWG	Yield ^a^ (%) of 9a-e	Yield ^a^ (%) of 11a-e
1	**8a**	MeOCO	66	59
2	**8b**	EtOCO	72	64
3	**8c**	BnOCO	62	53
4	**8d**	MeCO	57	47
5	**8e**	PhCO	54	45

^a^ Yields of purified isolated products.

The structures of all synthesized compounds were confirmed by ^1^H- and in some cases, ^13^C-NMR measurements. The ^1^H-NMR spectra of **6a**-**i** and **7a**-**i** revealed the appearance of the new signals of the incorporated aryl groups at 6.9-8.5 ppm as compared with the spectra of the starting azide **5**, while the 5’-H singlet of the newly-formed heterocycle was identified at 7.8-8.5 ppm. Compounds **6j** and **7j** containing a cycloalkyl substituent were exceptions, with a chemical shift of 7.28 ppm (5’-H). As far as the tetrazolyl derivatives are concerned, the newly-formed heterocycle does not contain any protons, but the signal of 5’-C can be identified at 145-149 ppm in the corresponding ^13^C-NMR spectra. Furthermore, in the cases of **9c**, **11c** and **9e**, **11e** the new signals of the incorporated Ph ring appeared at 7.3-8.3 ppm in the ^1^H-NMR spectra.

### 2.2. Biological Activity

Compounds **7a**-**j** and **11a**-**e** were screened for anticancer activity against a panel of three human cancer cell lines (Table 3). Although there is no generally accepted threshold for efficacy, when the inhibition of cell growth is less than 25% at 30 µM, such a substance may be considered ineffective. No clear structure-activity relationships could be concluded, but triazole-containing androst-5-enes exhibit substantial antiproliferative activity, for which a substituted aromatic group of the triazole ring is preferred. The antiproliferative action of a compound with an unsubstituted phenyl group on the triazole ring (compound **7a**) could be maintained or moderately increased by substitution in the *para* or *meta* position (compounds **7b**-**e**, **7g**), while *ortho*-OMe (compound **7f**) was less effective. Nevertheless, an amino group in the *meta* position offers no advantage (compound **7h**). A pyridyl, but not a cyclopropyl group, instead of phenyl (compounds **7i**-**j**) could be beneficial. Although inactive on HeLa cells at 10 µM, **7c** is considered the most effective of the presented compounds. In contrast, tetrazoles substituted on the D-ring of the steroidal skeleton proved to be ineffective with the exception of **11e**, which has a moderate effect.

**Table 3:** Antiproliferative effects of the synthesized compounds.

		Growth inhibition % (±SEM)					
Product	µM	HeLa		MCF7		A2780	
**7a**	10	64.6	(±1.6)	35.7	(±0.8)	<25 *	
	30	72.6	(±1.9)	37.2	(±2.2)	26.9	(±2.7)
**7b**	10	77.6	(±0.7)	40.8	(±1.8)	35.7	(±2.2)
	30	79.2	(±0.7)	54.8	(±2.6)	36.8	(±1.6)
**7c**	10	<25		41.6	(±0.2)	54.1	(±2.6)
	30	96.9	(±1.7)	83.7	(±1.3)	88.5	(±2.1)
**7d**	10	78.1	(±0.6)	47.0	(±2.1)	45.0	(±2.9)
	30	78.6	(±1.4)	47.6	(±1.6)	46.8	(±0.9)
**7e**	10	68.7	(±0.3)	60.2	(±1.9)	30.9	(±2.2)
	30	74.4	(±0.7)	62.6	(±0.7)	32.8	(±1.4)
**7f**	10	<25		<25		42.3	(±2.7)
	30	<25		27.1	(±1.3)	47.8	(±2.1)
**7g**	10	61.8	(±0.3)	54.5	(±1.1)	31.4	(±2.6)
	30	68.4	(±0.3)	59.3	(±1.8)	45.6	(±2.4)
**7h**	10	49.2	(±1.3)	<25		42.1	(±1.2)
	30	66.2	(±0.8)	28.6	(±2.4)	53.2	(±0.9)
**7i**	10	55.7	(±2.6)	46.5	(±2.1)	31.1	(±1.9)
	30	93.2	(±0.8)	63.8	(±1.0)	43.3	(±2.1)
**7j**	10	61.7	(±0.4)	31.3	(±1.1)	42.3	(±2.6)
	30	63.6	(±0.7)	50.5	(±1.1)	47.5	(±1.4)
**11a-d**	10	<25		<25		<25	
	30	<25		<25		<25	
**11e**	10	<25		<25		<25	
	30	<25		61.8	(±1.7)	72.0	(±0.4)
**Cisplatin**	10	42.6	(±2.3)	88.6	(±0.5)	53.0	(±2.3)
	30	99.9	(±0.3)	90.2	(±1.8)	86.9	(±1.2)

* Compounds eliciting less than 25% inhibition of proliferation were considered ineffective, and for simplicity the exact results are not given.

## 3. Experimental

### 3.1. General

Melting points (mp) were determined on a Kofler block and are uncorrected. The reactions were monitored by TLC on Kieselgel-G (Merck Si 254 F) layers (0.25 mm thick); solvent systems (ss): (A) CH_2_Cl_2_/EtOAc (95:5 v/v), (B) CH_2_Cl_2_/EtOAc (80:20 v/v), (C) CH_2_Cl_2_/EtOAc (50:50 v/v). The spots were detected by spraying with 5% phosphomolybdic acid in 50% aqueous phosphoric acid. The *R*_f_ values were determined for the spots observed by illumination at 254 and 365 nm. Flash chromatography: Merck silica gel 60, 40-63 μm. All solvents were distilled prior to use. Reagents and materials were obtained from commercial suppliers and were used without purification.

Elementary analysis data were determined with a PerkinElmer CHN analyzer model 2400 and IR spectra were recorded on a BioRad FTS 60A FTIR spectrometer. NMR spectra were obtained at room temperature with a Bruker DRX 500 instrument. Chemical shifts are reported in ppm (δ scale), and coupling constants (*J*) in Hz. For the determination of multiplicities, the *J*-MOD pulse sequence was used.

Automated flow injection analyses were performed by using an HPLC/MSD system. The system comprised an Agilent 1100 micro vacuum degasser, a quaternary pump, a micro-well plate autoinjector and a 1946A MSD equipped with an electrospray ion source (ESI) operated in positive ion mode. The ESI parameters were: nebulizing gas N_2_, at 35 psi; drying gas N_2_, at 350 °C and 12 L/min; capillary voltage (VCap) 3000 V; fragmentor voltage 70 V. The MSD was operated in scan mode with a mass range of *m/z* 60−620. Samples (0.2 μL) with automated needle wash were injected directly into the solvent flow (0.3 mL/min) of CH_3_CN/H_2_O, 70:30 (v/v) supplemented with 0.1% formic acid. The system was controlled by Agilent LC/MSD Chemstation software.

### 3.2. 3β-Acetoxy-16-acetoxymethylideneandrost-5-en-17-one (***2b***)

Compound **2a** (19.9 g, 63 mmol) was dissolved in a mixture of pyridine (40 mL) and Ac_2_O (40 mL), and the solution was stirred overnight, and then poured onto a mixture of ice and H_2_SO_4_ (18 mL). The precipitate was collected by filtration, washed to neutrality and dried, resulting in 23.8 g (94%) of **2b**, mp 199-202 °C (lit. [28] mp 198-200 °C), *R*_f_ = 0.68 (ss A).

### 3.3. 3β-Acetoxy-16β-hydroxymethylandrost-5-en-17β-ol (***3a***)

Finely powdered **2b** (23.8 g, 59.5 mmol) was suspended in a mixture of MeOH and EtOH (1:1, 500 mL), and KBH_4_ (8 g, 148 mmol) was added in small portions. To maintain pH 6-8, the solution was repeatedly acidified as needed with MeOH/AcOH (1:1), using bromothymol blue as indicator. After completion of the reaction, the mixture was diluted with water and acidified with dilute HCl. The precipitate that formed was filtered off and washed with water to neutrality. The resulting crude product was purified by column chromatography, with CH_2_Cl_2_/EtOAc (8:2) as eluent, yielding **3a **as a white solid (10.35 g, 48%), mp 197-199 °C (lit. [28] mp 199-201 °C), *R*_f_ = 0.44 (ss C). The spectroscopic data were consistent with those reported in the literature.

### 3.4. 3β-Acetoxy-16β-p-toluenesulfonyloxymethylandrost-5-en-17β-ol (***4***)

Compound **3a** (7.25 g, 20 mmol) was dissolved in pyridine (50 mL), and a solution of *p*-toluene-sulfonyl chloride (7 g, 35 mmol) in pyridine (10 mL) was then added dropwise while cooling in ice. The reaction mixture was allowed to stand overnight, and was then poured into a mixture of ice and H_2_SO_4 _(20 mL). The precipitate that formed was filtered off and washed with water to neutrality. This substance was used in the subsequent step without further purification and characterization. 

### 3.5. 3β-Acetoxy-16β-azidomethylandrost-5-en-17β-ol (***5***)

Sodium azide (1.8 g, 28 mmol) was added to a solution of **4** (5.8 g, 11 mmol) in DMF (80 mL). The reaction mixture was stirred at 70 °C for 6 h and was then poured into water. The precipitate that formed was allowed to stand overnight, and then filtered off and washed with water. Purification of the resulting crude product by column chromatography with CH_2_Cl_2_ as eluent afforded **5** as a white solid (3.75 g, 86%), mp 144-145 °C, *R*_f_ = 0.58 (ss A); ^1^H-NMR (CDCl_3_); δ [ppm] = 0.78 (s, 3H, 18-CH_3_), 0.95 (m, 1H), 1.03 (s, 3H, 19-CH_3_), 1.08-1.17 (overlapping m, 3H), 1.45 (m, 1H), 1.51-1.62 (overlapping m, 5H), 1.82-1.90 (overlapping m, 4H), 1.99 (m, 1H), 2.03 (s, 3H, Ac-CH_3_), 2.32 (m, 2H, 4-H_2_), 2.38 (m, 1H, 16-H), 3.31 (dd, 1H, *J* = 12.0 Hz, *J* = 6.5 Hz, 16a-H), 3.57 (dd, 1H, *J* = 12.0 Hz, *J* = 7.0 Hz, 16a-H), 3.79 (d, 1H, *J* = 10.0 Hz, 17-H), 4.60 (m, 1H, 3-H), 5.37 (d, 1H, *J* = 5.0 Hz, 6-H);^13^C-NMR (CDCl_3_); δ [ppm] = 12.1 (C-18), 19.3 (C-19), 20.5 (C-11), 21.4 (Ac-CH_3_), 27.7, 30.5, 31.1, 31.6, 36.6, 37.0, 37.4, 38.0, 39.9, 43.6, 49.9, 50.0, 53.3, 73.8 (C-3), 81.3 (C-17), 122.1 (C-6), 139.7 (C-5), 170.5 (Ac-CO); IR (neat, cm^−1^) 3526, 2945, 2909, 2112, 1717, 1439, 1365, 1256, 1032. ESI-MS: 388 (M+H)^+^; Anal. Calcd for C_22_H_33_N_3_O_3_ C, 68.19; H, 8.58; N, 10.84. Found: C, 68.01; H, 8.73; N, 11.04.

### 3.6. General Procedure for Preparation of 3β-acetoxy-16β-(4-phenyl-, substituted 4-phenyl- or 4-cycloalkyl-1H-1,2,3-triazol-1-ylmethyl)androst-5-en-17β-ols ***6a-j***

Compound **5** (387 mg, 1 mmol) was dissolved in CH_2_Cl_2_ (10 mL), and a solution of CuSO_4_·5H_2_O (12.5 mg, 5 mol %) and sodium ascorbate (30 mg, 15 mol %) in water (10 mL) was poured into the organic phase. The appropriate terminal alkyne (1.1 mmol) was added to the reaction mixture, which was then stirred for 1-4 h at ambient temperature. After the disappearance of the starting material (TLC monitoring), the two-phase solution was diluted with water (20 mL) and extracted with CH_2_Cl_2_ (2 × 20 mL). The combined organic layers were washed with water, dried over Na_2_SO_4_ and evaporated *in vacuo.* The resulting crude product was purified by flash chromatography with CH_2_Cl_2_/EtOAc (90:10), or CH_2_Cl_2_/EtOAc (80:20) as eluent.

*3β-Acetoxy-16β-(4-phenyl-1H-1,2,3-triazol-1-ylmethyl)androst-5-en-17β-ol* (**6a**): Alkyne: phenyl-acetylene (0.12 mL). After purification, **6a** was obtained as a white solid (435 mg, 89%), mp 251-252 °C, *R*_f_ = 0.30 (ss B); ^1^H-NMR (CDCl_3_); δ [ppm] = 0.80 (s, 3H, 18-CH_3_), 0.89 (m, 1H), 1.02 (s, 3H, 19-CH_3_), 1.11-1.20 (overlapping m, 3H), 1.37-1.59 (overlapping m, 6H), 1.80-1.91 (overlapping m, 4H), 1.97 (dd, 1H, *J* = 12.0 Hz, *J* = 2.5 Hz), 2.03 (s, 3H, Ac-CH_3_), 2.32 (m, 2H, 4-H_2_), 2.74 (m, 1H, 16-H), 3.88 (d, 1H, *J* = 10.0 Hz, 17-H), 4.32 (dd, 1H, *J* = 13.5 Hz, *J* = 6.5 Hz, 16a-H), 4.58 (m, 1H, 3-H), 4.70 (dd, 1H, *J* = 13.5 Hz, *J* = 7.0 Hz, 16a-H), 5.34 (br s, 1H, 6-H), 7.32 (t, 1H, *J* = 7.0 Hz, 4”-H), 7.41 (t, 2H, *J* = 7.0 Hz, 3”- and 5”-H), 7.82 (m, 3H, 2”-, 6”- and 5’-H); ^13^C-NMR (CDCl_3_); δ [ppm] = 12.3 (C-18), 19.3 (C-19), 20.5 (C-11), 21.4 (Ac-CH_3_), 27.7, 31.0, 31.1, 31.6, 36.6, 37.0, 37.3, 38.0, 41.3, 43.7, 49.9, 50.0, 52.0, 73.8 (C-3), 80.6 (C-17), 120.6 (C-5’), 122.0 (C-6), 125.7 (2C, C-2” and C-6”), 128.2 (C-4”), 128.8 (2C, C-3” and C-5”), 130.2 (C-1”), 139.7 (C-5), 147.2 (C-4’), 170.6 (Ac-CO); IR (neat, cm^−1^) 3404, 2941, 2910, 1732, 1373, 1242, 1034, 772, 706. ESI-MS: 490 (M+H)^+^; Anal. Calcd for C_30_H_39_N_3_O_3_: C, 73.59; H, 8.03; N, 8.58. Found: C, 73.70; H, 8.19; N, 8.76.

*3β-Acetoxy-16β-[4-(4-ethylphenyl)-1H-1,2,3-triazol-1-ylmethyl]androst-5-en-17β-ol* (**6b**): Alkyne: 4-ethylphenylacetylene (0.15 mL). After purification, **6b** was obtained as a white solid (470 mg, 91%), mp 249-250 °C, *R*_f_ = 0.36 (ss B); ^1^H-NMR (CDCl_3_); δ [ppm] = 0.79 (s, 3H, 18-CH_3_), 0.94 (m, 1H), 1.02 (s, 3H, 19-CH_3_), 1.09-1.21 (overlapping m, 3H), 1.25 (t, 3H, *J* = 7.5 Hz, Et-CH_3_), 1.41-1.62 (overlapping m, 6H), 1.79-1.92 (overlapping m, 4H), 1.95 (dd, 1H, *J* = 12.5 Hz, *J* = 2.5 Hz), 2.03 (s, 3H, Ac-CH_3_), 2.32 (m, 2H, 4-H_2_), 2.67 (q, 2H, *J* = 7.5 Hz, Et-CH_2_), 2.73 (m, 1H, 16-H), 3.87 (d, 1H, *J* = 10.0 Hz, 17-H), 4.30 (dd, 1H, *J* = 13.5 Hz, *J* = 6.5 Hz, 16a-H), 4.58 (m, 1H, 3-H), 4.69 (dd, 1H, *J* = 13.5 Hz, *J* = 7.0 Hz, 16a-H), 5.34 (br s, 1H, 6-H), 7.24 (d, 2H, *J* = 7.5 Hz, 3”- and 5”-H), 7.72 (d, 2H, *J* = 7.5 Hz, 2”- and 6”-H), 7.79 (s, 1H, 5’-H); ^13^C-NMR (CDCl_3_); δ [ppm] = 12.3 (C-18), 19.3 (C-19), 20.5 (C-11), 21.4 (Ac-CH_3_), 27.7, 28.6, 31.0, 31.1, 31.6, 36.6, 36.9, 37.0, 37.3, 38.0, 41.3, 43.7, 49.9, 50.0, 52.0, 73.8, 80.6, 120.3 (C-5’), 122.1 (C-6), 125.7 (2C, C-2” and C-6”), 127.7 (C-1”), 128.3 (2C, C-3” and C-5”), 139.7 (C-5), 144.4 (C-4”), 147.3 (C-4’), 170.5 (Ac-CO); IR (neat, cm^−1^) 3383, 2945, 2854, 1730, 1375, 1248, 1032, 976, 833, 613. ESI-MS: 518 (M+H)^+^; Anal. Calcd for C_32_H_43_N_3_O_3_: C, 74.24; H, 8.37; N, 8.12. Found: C, 74.38; H, 8.24; N, 8.19.

*3β-Acetoxy-16β-[4-(3-tolyl)-1H-1,2,3-triazol-1-ylmethyl]androst-5-en-17β-ol* (**6c**): Alkyne: 3-tolyl-acetylene (0.14 mL). After purification, **6c **was obtained as a white solid (458 mg, 91%), mp 232-233 °C, *R*_f_ = 0.36 (ss B); ^1^H-NMR (CDCl_3_); δ [ppm] = 0.79 (s, 3H, 18-CH_3_), 0.95 (m, 1H), 1.01 (s, 3H, 19-CH_3_), 1.08-1.21 (overlapping m, 3H), 1.33-1.61 (overlapping m, 6H), 1.79-1.90 (overlapping m, 4H), 1.95 (dd, 1H, *J* = 12.0 Hz, *J* = 3.0 Hz), 2.02 (s, 3H, Ac-CH_3_), 2.31 (m, 2H, 4-H_2_), 2.38 (s, 3H, 3”-CH_3_), 2.72 (m, 1H, 16-H), 3.87 (d, 1H, *J* = 10.0 Hz, 17-H), 4.30 (dd, 1H, *J* = 13.5 Hz, *J* = 6.5 Hz, 16a-H), 4.58 (m, 1H, 3-H), 4.69 (dd, 1H, *J* = 14.0 Hz, *J* = 6.5 Hz, 16a-H), 5.33 (d, 1H, *J* = 4.0 Hz, 6-H), 7.13 (d, 1H, *J* = 7.0 Hz, 4”-H), 7.29 (t, 1H, *J* = 7.5 Hz, 5”-H), 7.57 (d, 1H, *J* = 7.5 Hz, 6”-H), 7.65 (s, 1H, 2”-H), 7.81 (s, 1H, 5’-H); ^13^C-NMR (CDCl_3_); δ [ppm] = 12.3 (C-18), 19.3 (C-19), 20.4 (C-11), 21.4 (Ac-CH_3_), 27.7, 29.6, 31.0, 31.1, 31.6, 36.6, 37.0, 37.3, 38.0, 41.3, 43.7, 49.8, 50.0, 52.0, 73.8 (C-3), 80.6 (C-17), 120.6 (C-5’), 122.0 (C-6), (122.7, 126.3, 128.7, 128.9): (4C, C-2”, C-4”, C-5” and C-6”), 130.2 (C-1”), 138.5 (C-3”), 139.7 (C-5), 147.3 (C-4’), 170.6 (Ac-CO); IR (neat, cm^−1^) 3406, 2922, 2850, 1731, 1364, 1244, 1024, 787, 696. ESI-MS: 504 (M+H)^+^; Anal. Calcd for C_31_H_41_N_3_O_3_: C, 73.92; H, 8.20; N, 8.34. Found: C, 73.80; H, 8.38; N, 8.49.

*3β-Acetoxy-16β-[4-(4-tert-butylphenyl)-1H-1,2,3-triazol-1-ylmethyl]androst-5-en-17β-ol* (**6d**): Alkyne: 4-*tert*-butylphenylacetylene (0.2 mL). After purification, **6d **was obtained as a white solid (507 mg, 93%), mp 318-319 °C, *R*_f_ = 0.39 (ss B); ^1^H-NMR (CDCl_3_); δ [ppm] = 0.79 (s, 3H, 18-CH_3_), 0.88 (m, 1H), 1.02 (s, 3H, 19-CH_3_), 1.34 (s, 9H, 3 x *t*Bu-CH_3_), 1.11-1.20 (overlapping m, 3H), 1.37-1.60 (overlapping m, 6H), 1.79-1.91 (overlapping m, 4H), 1.96 (dd, 1H, *J* = 11.5 Hz, *J* = 2.5 Hz), 2.03 (s, 3H, Ac-CH_3_), 2.31 (m, 2H, 4-H_2_), 2.75 (m, 1H, 16-H), 3.89 (d, 1H, *J* = 10.0 Hz, 17-H), 4.31 (m, 1H, 16a-H), 4.60 (m, 1H, 3-H), 4.72 (dd, 1H, *J* = 13.5 Hz, *J* = 6.5 Hz, 16a-H), 5.34 (d, 1H, *J* = 4.0 Hz, 6-H), 7.45 (d, 2H, *J* = 6.0 Hz, 3”- and 5”-H), 7.78 (d, 2H, *J* = 6.0 Hz, 2”- and 6”-H), 7.87 (s, 1H, 5’-H); ^13^C-NMR (CDCl_3_); δ [ppm] = 12.3 (C-18), 19.3 (C-19), 20.5 (C-11), 21.4 (Ac-CH_3_), 27.7, 29.7, 30.9, 31.1, 31.3 (3C, 3 x *t*Bu-CH_3_), 31.6, 36.6, 37.0, 37.3, 38.0, 41.3, 43.8, 49.9, 50.0, 52.1, 73.7 (C-3), 80.6 (C-17), 120.4 (C-5’), 122.0 (C-6), 125.5 (2C), 125.8 (2C), 127.3 (C-1”), 139.7 (C-5), 147.1 (C-4’), 151.4 (C-4”), 170.5 (Ac-CO); IR (neat, cm^−1^) 3441, 2949, 2903, 1730, 1454, 1366, 1247, 1034, 837, 810, 557. ESI-MS: 546 (M+H)^+^; Anal. Calcd for C_34_H_47_N_3_O_3_: C, 74.83; H, 8.68; N, 7.70. Found: C, 75.02; H, 8.61; N, 7.82.

*3β-Acetoxy-16β-[4-(4-methoxyphenyl)-1H-1,2,3-triazol-1-ylmethyl]androst-5-en-17β-ol* (**6e**): Alkyne: 4-methoxyphenylacetylene (145 mg). After purification, **6e **was obtained as a white solid (447 mg, 86%), mp 241-242 °C, *R*_f_ = 0.34 (ss B); ^1^H-NMR (500 MHz, 10% MeOD/CDCl_3_); δ [ppm] = 0.81 (s, 3H, 18-CH_3_), 0.93 (m, 1H), 1.03 (s, 3H, 19-CH_3_), 1.09-1.21 (overlapping m, 3H), 1.33-1.62 (overlapping m, 6H), 1.77-1.90 (overlapping m, 4H), 1.97 (dd, 1H, *J* = 12.0 Hz, *J* = 2.5 Hz), 2.03 (s, 3H, Ac-CH_3_), 2.32 (m, 2H, 4-H_2_), 2.72 (m, 1H, 16-H), 3.82 (d, 1H, *J* = 10.0 Hz, 17-H), 3.86 (s, 3H, OCH_3_), 4.28 (dd, 1H, *J* = 13.5 Hz, *J* = 8.0 Hz, 16a-H), 4.58 (m, 1H, 3-H), 4.70 (dd, 1H, *J* = 13.5 Hz, *J* = 6.0 Hz, 16a-H), 5.34 (br s, 1H, 6-H), 6.96 (d, 2H, *J* = 8.5 Hz, 3”- and 5”-H), 7.21 (d, 2H, *J* = 8.5 Hz, 2”- and 6”-H), 7.81 (s, 1H, 5’-H); IR (neat, cm^−1^) 3398, 2935, 2902, 1728, 1499, 1373, 1244, 1063, 839, 538. ESI-MS: 520 (M+H)^+^; Anal. Calcd for C_31_H_41_N_3_O_4_: C, 71.65; H, 7.95; N, 8.09. Found: C, 71.74; H, 7.76; N, 8.22.

*3β-Acetoxy-16β-[4-(2-methoxyphenyl)-1H-1,2,3-triazol-1-ylmethyl]androst-5-en-17β-ol* (**6f**): Alkyne: 2-methoxyphenylacetylene (0.14 mL). After purification, **6f** was obtained as a white solid (430 mg, 83%), mp 278-279 °C, *R*_f_ = 0.32 (ss B); ^1^H-NMR (CDCl_3_); δ [ppm] = 0.79 (s, 3H, 18-CH_3_), 0.94 (m, 1H), 1.01 (s, 3H, 19-CH_3_), 1.08-1.20 (overlapping m, 3H), 1.33-1.60 (overlapping m, 6H), 1.79-1.96 (overlapping m, 5H), 2.02 (s, 3H, Ac-CH_3_), 2.31 (m, 2H, 4-H_2_), 2.73 (m, 1H, 16-H), 3.87 (d, 1H, *J* = 10.0 Hz, 17-H), 3.92 (s, 3H, OCH_3_), 4.30 (dd, 1H, *J* = 13.5 Hz, *J* = 6.5 Hz, 16a-H), 4.58 (m, 1H, 3-H), 4.70 (dd, 1H, *J* = 13.5 Hz, *J* = 7.0 Hz, 16a-H), 5.34 (br s, 1H, 6-H), 6.95 (d, 1H, *J* = 8.0 Hz, 3”-H), 7.06 (t, 1H, *J* = 7.5 Hz, 5”-H), 7.29 (t, 1H, *J* = 7.5 Hz, 4”-H), 8.05 (s, 1H, 5’-H), 8.31 (d, 1H, *J* = 7.5 Hz, 6”-H); ^13^C-NMR (CDCl_3_); δ [ppm] = 12.3 (C-18), 19.3 (C-19), 20.5 (C-11), 21.4 (Ac-CH_3_), 27.7, 31.0, 31.1, 31.6, 36.6, 37.0, 37.3, 38.0, 41.3, 43.7, 49.9, 50.0, 51.8, 55.3 (OCH_3_), 73.8 (C-3), 80.6 (C-17), 110.7, 119.2 (C-1”), 120.9 (C-5’), 122.0 (C-6), 123.9, 127.5, 128.8, 139.7 (C-5), 142.7 (C-4’), 155.5 (C-2”), 170.6 (Ac-CO); IR (neat, cm^−1^) 3400, 2939, 2907, 1732, 1491, 1373, 1246, 1060, 974, 750, 679. ESI-MS: 520 (M+H)^+^; Anal. Calcd for C_31_H_41_N_3_O_4_: C, 71.65; H, 7.95; N, 8.09. Found: C, 71.76; H, 8.08; N, 8.01.

*3β-Acetoxy-16β-[4-(4-fluorophenyl)-1H-1,2,3-triazol-1-ylmethyl]androst-5-en-17β-ol* (**6g**): Alkyne: 4-fluorophenylacetylene (0.13 mL). After purification, **6g** was obtained as a white solid (430 mg, 85%), mp 263-264 °C, *R*_f_ = 0.36 (ss B); ^1^H-NMR (10% MeOD/CDCl_3_); δ [ppm] = 0.82 (s, 3H, 18-CH_3_), 0.96 (m, 1H), 1.03 (s, 3H, 19-CH_3_), 1.10-1.22 (overlapping m, 3H), 1.40-1.63 (overlapping m, 6H), 1.78-1.91 (overlapping m, 4H), 1.97 (dd, 1H, *J* = 13.0 Hz, *J* = 2.5 Hz), 2.04 (s, 3H, Ac-CH_3_), 2.32 (m, 2H, 4-H_2_), 2.72 (m, 1H, 16-H), 3.84 (d, 1H, *J* = 10.0 Hz, 17-H), 4.30 (dd, 1H, *J* = 13.5 Hz, *J* = 8.0 Hz, 16a-H), 4.58 (m, 1H, 3-H), 4.71 (dd, 1H, *J* = 13.5 Hz, *J* = 6.0 Hz, 16a-H), 5.34 (br s, 1H, 6-H), 7.11 (t, 2H, *J* = 7.5 Hz, 3”- and 5”-H), 7.76 (t, 2H, *J* = 7.5 Hz, 2”- and 6”-H), 7.88 (s, 1H, 5’-H); ^13^C-NMR (10% MeOD/CDCl_3_); δ [ppm] = 12.1 (C-18), 19.1 (C-19), 20.3 (C-11), 21.2 (Ac-CH_3_), 27.5, 30.9, 31.0, 31.4, 36.5, 36.8, 37.1, 37.8, 41.0, 43.5, 49.8, 49,9, 52.4, 73.9 (C-3), 80.3 (C-17), 115.7 (d, 2C, *J* = 21.5 Hz, C-3” and C-5”), 120.4 (C-5’), 122.0 (C-6), 126.5 (C-1”), 127.2 (d, 2C, *J* = 8 Hz, C-2” and C-6”), 139.5 (C-5), 146.4 (C-4’), 162.5 (d, *J* = 245 Hz, C-4”), 170.9 (Ac-CO); IR (neat, cm^−1^) 3412, 2945, 2912, 1730, 1460, 1243, 1062, 812, 524. ESI-MS: 508 (M+H)^+^; Anal. Calcd for C_30_H_38_FN_3_O_3_: C, 70.98; H, 7.55; N, 8.28. Found: C, 70.86; H, 7.64; N, 8.43.

*3β-Acetoxy-16β-[4-(3-aminophenyl)-1H-1,2,3-triazol-1-ylmethyl]androst-5-en-17β-ol* (**6h**): Alkyne: 3-aminophenylacetylene (0.12 mL). After purification, **6h** was obtained as a white solid (454 mg, 90%), mp 255-256 °C, *R*_f_ = 0.30 (ss C); ^1^H-NMR (DMSO-*d_6_*); δ [ppm] = 0.76 (s, 3H, 18-CH_3_), 0.91 (m, 2H), 0.98 (s, 3H, 19-CH_3_), 1.02-1.15 (overlapping m, 3H), 1.38-1.56 (overlapping m, 6H), 1.74-1.90 (overlapping m, 4H), 1.97 (s, 3H, Ac-CH_3_), 2.25 (m, 2H, 4-H_2_), 2.64 (m, 1H, 16-H), 3.71 (d, 1H, *J* = 10.0 Hz, 17-H), 4.17 (t, 1H, *J* = 12.5 Hz, 16a-H), 4.43 (m, 1H, 3-H), 4.57 (dd, 1H, *J* = 13.5 Hz, *J* = 5.0 Hz, 16a-H), 5.02 (br s, 1H, OH), 5.30 (br s, 1H, 6-H), 5.66 (br s, 2H, NH_2_), 6.56 (d, 1H, *J* = 7.0 Hz, 4”-H), 6.98 (d, 1H, *J* = 7.0 Hz, 6”-H), 7.08 (t, 1H, *J* = 7.5 Hz, 5”-H), 7.14 (s, 1H, 2”-H), 8.41 (s, 1H, 5’-H); ^13^C-NMR (DMSO-*d_6_*); δ [ppm] = 12.4 (C-18), 19.0 (C-19), 20.1 (C-11), 21.0 (Ac-CH_3_), 27.3, 30.4, 30.7, 31.0, 36.1, 36.4, 36.8, 37.6, 40.3, 43.2, 49.1, 49.6, 52.2, 73.1 (C-3), 79.4 (C-17), 110.9, 113.7, 113.9, 121.1, 121.9, 129.3, 131.4, 139.4, 146.5, 147.8, 169.7 (Ac-CO); IR (neat, cm^−1^) 3340, 3228, 2941, 1732, 1454, 1242, 1069, 1034, 795. ESI-MS: 505 (M+H)^+^; Anal. Calcd for C_30_H_40_N_4_O_3_: C, 71.40; H, 7.99; N, 11.10. Found: C, 71.54; H, 7.86; N, 11.23.

*3β-Acetoxy-16β-[4-(2-pyridyl)-1H-1,2,3-triazol-1-ylmethyl]androst-5-en-17β-ol* (**6i**): Alkyne: 2 pyridylacetylene (0.11 mL). After purification, **6i **was obtained as a white solid (427 mg, 87%), mp 259-260 °C, *R*_f_ = 0.23 (ss C); ^1^H-NMR (CDCl_3_); δ [ppm] = 0.80 (s, 3H, 18-CH_3_), 0.93 (m, 1H), 1.01 (s, 3H, 19-CH_3_), 1.16-1.27 (overlapping m, 3H), 1.39-1.59 (overlapping m, 6H), 1.77-1.87 (overlapping m, 4H), 1.94 (dd, 1H, *J* = 12.5 Hz, *J* = 2.5 Hz), 2.01 (s, 3H, Ac-CH_3_), 2.30 (m, 2H, 4-H_2_), 2.72 (m, 1H, 16-H), 3.87 (d, 1H, *J* = 10.0 Hz, 17-H), 4.34 (dd, 1H, *J* = 13.5 Hz, *J* = 7.5 Hz, 16a-H), 4.57 (m, 1H, 3-H), 4.75 (dd, 1H, *J* = 13.5 Hz, *J* = 6.5 Hz, 16a-H), 5.32 (d, 1H, *J* = 3.0 Hz, 6-H), 7.24 (m, 1H, 4”-H), 7.79 (t, 1H, *J* = 7.5 Hz, 5”-H), 8.17 (d, 1H, *J* = 7.5 Hz, 6”-H), 8.30 (s, 1H, 5’-H), 8.54 (d, 1H, *J* = 3.0 Hz, 3”-H); ^13^C-NMR (CDCl_3_); δ [ppm] = 12.3 (C-18), 19.3 (C-19), 20.4 (C-11), 21.4 (Ac-CH_3_), 27.7, 31.0, 31.1, 31.6, 36.6, 37.0, 37.3, 38.0, 41.3, 43.7, 49.8, 50.0, 52.4, 73.8 (C-3), 80.5 (C-17), 120.4 (C-5’), 122.1 (C-6), 122.8, 123.1, 137.4, 139.6 (C-5), 147.3 (C-2”), 148.8 (C-6”), 149.9 (C-4’), 170.5 (Ac-CO); IR (neat, cm^−1^) 3395, 2932, 2911, 1731, 1435, 1364, 1240, 1032, 789, 540. ESI-MS: 491 (M+H)^+^; Anal. Calcd for C_29_H_38_N_4_O_3_: C, 70.99; H, 7.81; N, 11.42. Found: C, 71.16; H, 7.97; N, 11.61.

*3β-Acetoxy-16β-(4-cyclopropyl-1H-1,2,3-triazol-1-ylmethyl)androst-5-en-17β-ol* (**6j**): Alkyne: cyclo-propylacetylene (0.09 mL). After purification, **6j **was obtained as a white solid (355 mg, 78%), mp 261-263 °C, *R*_f_ = 0.30 (ss B); ^1^H-NMR (CDCl_3_); δ [ppm] = 0.76 (s, 3H, 18-CH_3_), 0.83 (m, 2H), 0.90-0.96 (overlapping m, 4H), 1.01 (s, 3H, 19-CH_3_), 1.08-1.17 (overlapping m, 3H), 1.41-1.61 (overlapping m, 5H), 1.76-1.95 (overlapping m, 5H), 2.02 (s, 3H, Ac-CH_3_), 2.31 (m, 2H, 4-H_2_), 2.63 (m, 1H, 16-H), 3.08 (br s, 1H, 1”-H), 3.84 (dd, 1H, *J* = 10.0 Hz, *J* = 3.5 Hz, 17-H), 4.19 (dd, 1H, *J* = 13.5 Hz, *J* = 6.5 Hz, 16a-H), 4.57-4.61 (overlapping m, 2H, 3- and 16a-H), 5.33 (d, 1H, *J* = 3.5 Hz, 6-H), 7.28 (s, 1H, 5’-H); ^13^C-NMR (CDCl_3_); δ [ppm] = 6.6, 7.7 (2C), 12.3 (C-18), 19.3 (C-19), 20.5 (C-11), 21.4 (Ac-CH_3_), 27.7, 31.0, 31.1, 31.6, 36.6, 37.0, 37.3, 38.0, 41.4, 43.7, 49.9, 50.0, 51.7, 73.8 (C-3), 80.5 (C-17), 120.5 (C-5’), 122.1 (C-6), 139.7 (C-5), 149.9 (C-4’), 170.5 (Ac-CO); IR (neat, cm^−1^) 3394, 2943, 1732, 1431, 1372, 1246, 1068, 1034, 814. ESI-MS: 454 (M+H)^+^; Anal. Calcd for C_27_H_39_N_3_O_3_: C, 71.49; H, 8.67; N, 9.26. Found: C, 71.61; H, 8.82; N, 9.57.

### 3.7. General procedure for preparation of 16β-(4-phenyl-, substituted 4-phenyl- or 4-cycloalkyl-1H-1,2,3-triazol-1-ylmethyl)androst-5-ene-3β,17β-diols ***7a-j***

Compounds **6a**-**j** (0.5 mmol) were deacetylated by dissolving in MeOH (20 mL), adding KOH (150 mg, 2.7 mmol), stirring the mixture for 1 h at 50 °C, and then pouring into water (200 mL) and neutralizing with diluted HCl. The resulting precipitate was filtered off, washed with water and dried. The crude product obtained was purified by flash chromatography (silica gel) to afford **7a**-**j**.

*16β-(4-Phenyl-1H-1,2,3-triazol-1-ylmethyl)androst-5-ene-3β,17β-diol* (**7a**): Eluent: CH_2_Cl_2_/EtOAc (75:25), yielding **7a **as a white solid (184 mg, 82%), mp 264-265 °C, *R*_f_ = 0.45 (ss C); ^1^H-NMR (10% MeOD/CDCl_3_); δ [ppm] = 0.82 (s, 3H, 18-CH_3_), 0.94-0.98 (m, 2H), 1.02 (s, 3H, 19-CH_3_), 1.09-1.25 (overlapping m, 3H), 1.45-1.61 (overlapping m, 5H), 1.81-1.97 (overlapping m, 5H), 2.20-2.28 (m, 2H), 2.73 (m, 1H, 16-H), 3.47 (m, 1H, 3-H), 3.84 (d, 1H, *J* = 10.0 Hz, 17-H), 4.30 (dd, 1H, *J* = 13.5 Hz, *J* = 8.0 Hz, 16a-H), 4.72 (dd, 1H, *J* = 13.5 Hz, *J* = 6.0 Hz, 16a-H), 5.31 (br s, 1H, 6-H), 7.34 (t, 1H, *J* = 7.5 Hz, 4”-H), 7.43 (t, 2H, *J* = 7.5 Hz, 3”- and 5”-H), 7.79 (d, 2H, *J* = 7.5 Hz, 2”- and 6”-H), 7.89 (s, 1H, 5’-H); IR (neat, cm^−1^) 3428, 2944, 2904, 1444, 1236, 1080, 827, 760, 691. ESI-MS: 448 (M+H)^+^; Anal. Calcd for C_28_H_37_N_3_O_2_: C, 75.13; H, 8.33; N, 9.39. Found: C, 75.27; H, 8.21; N, 9.56.

*16β-[4-(4-Ethylphenyl)-1H-1,2,3-triazol-1-ylmethyl]androst-5-ene-3β,17β-diol* (**7b**): Eluent: CH_2_Cl_2_/ EtOAc (75:25), yielding **7b **as a white solid (192 mg, 81%), mp 261-262 °C, *R*_f_ = 0.48 (ss C); ^1^H-NMR (DMSO-*d_6_*); δ [ppm] = 0.75 (s, 3H, 18-CH_3_), 0.83-0.88 (m, 2H), 0.94 (s, 3H, 19-CH_3_), 1.03-1.12 (overlapping m, 3H), 1.19 (t, 3H, *J* = 7.5 Hz, Et-CH_3_), 1.30-1.44 (overlapping m, 4H), 1.52 (m, 2H), 1.66 (m, 1H), 1.74-1.86 (overlapping m, 3H), 2.05-2.14 (m, 2H), 2.59 (q, 2H, *J* = 7.5 Hz, Et-CH_2_), 2.65 (m, 1H, 16-H), 3.24 (m, 1H, 3-H), 3.71 (dd, 1H, *J* = 9.5 Hz, *J* = 3.5 Hz, 17-H), 4.18 (m, 1H, 16a-H), 4.58 (overlapping m, 2H, 3-OH and 16a-H), 5.01 (d, 1H, *J* = 3.5 Hz, 17-OH), 5.21 (br s, 1H, 6-H), 7.26 (d, 2H, *J* = 7.5 Hz, 3”- and 5”-H), 7.74 (d, 2H, *J* = 7.5 Hz, 2”- and 6”-H), 8.51 (s, 1H, 5’-H); IR (neat, cm^−1^) 3383, 2943, 1440, 1240, 1082, 1051, 812, 738, 644. ESI-MS: 476 (M+H)^+^; Anal. Calcd for C_30_H_41_N_3_O_2_: C, 75.75; H, 8.69; N, 8.83. Found: C, 75.91; H, 8.87; N, 8.70.

*16β-[4-(3-Tolyl)-1H-1,2,3-triazol-1-ylmethyl]androst-5-ene-3β,17β-diol* (**7c**): Eluent: CH_2_Cl_2_/EtOAc (75:25), yielding **7c **as a white solid (203 mg, 88%), mp 237-238 °C, *R*_f_ = 0.44 (ss C); ^1^H-NMR (DMSO-*d_6_*); δ [ppm] = 0.76 (s, 3H, 18-CH_3_), 0.86 (m, 2H), 0.94 (s, 3H, 19-CH_3_), 1.04-1.17 (overlapping m, 3H), 1.31-1.43 (overlapping m, 4H), 1.51 (m, 2H), 1.65 (m, 1H), 1.73-1.87 (overlapping m, 3H), 2.07-2.15 (m, 2H), 2.34 (s, 3H, 3”-CH_3_), 2.64 (m, 1H, 16-H), 3.24 (m, 1H, 3-H), 3.71 (dd, 1H, *J* = 9.5 Hz, *J* = 4.0 Hz, 17-H), 4.19 (m, 1H, 16a-H), 4.59 (overlapping m, 2H, 3-OH and 16a-H), 5.00 (d, 1H, *J* = 4.0 Hz, 17-OH), 5.21 (br s, 1H, 6-H), 7.12 (d, 1H, *J* = 7.0 Hz, 4”-H), 7.31 (t, 1H, *J* = 7.5 Hz, 5”-H), 7.62 (d, 1H, *J* = 7.0 Hz, 6”-H), 7.66 (s, 1H, 2”-H), 8.53 (s, 1H, 5’-H); IR (neat, cm^−1^) 3339, 3237, 2931, 1452, 1232, 1049, 787, 696. ESI-MS: 462 (M+H)^+^; Anal. Calcd for C_29_H_39_N_3_O_2_: C, 75.45; H, 8.52; N, 9.10. Found: C, 75.57; H, 8.67; N, 9.32.

*16β-[4-(4-Tert-butylphenyl)-1H-1,2,3-triazol-1-ylmethyl]androst-5-ene-3β,17β-diol* (**7d**): Eluent: CH_2_Cl_2_/EtOAc (75:25), yielding **7d **as a white solid (214 mg, 85%), mp 284-285 °C, *R*_f_ = 0.49 (ss C); ^1^H-NMR (10% MeOD/CDCl_3_); δ [ppm] = 0.75 (s, 3H, 18-CH_3_), 0.85-0.89 (m, 2H), 0.94 (s, 3H, 19-CH_3_), 1.27 (s, 9H, 3 x *t*Bu-CH_3_), 0.99-1.13 (overlapping m, 3H), 1.40-1.53 (overlapping m, 5H), 1.70-1.89 (overlapping m, 5H), 2.14-2.21 (m, 2H), 2.66 (m, 1H, 16-H), 3.40 (m, 1H, 3-H), 3.76 (d, 1H, *J* = 10.0 Hz, 17-H), 4.21 (dd, 1H, *J* = 14.0 Hz, *J* = 8.5 Hz, 16a-H), 4.64 (dd, 1H, *J* = 13.5 Hz, *J* = 6.0 Hz, 16a-H), 5.24 (br s, 1H, 6-H), 7.38 (d, 2H, *J* = 8.0 Hz, 3”- and 5”-H), 7.64 (d, 2H, *J* = 8.0 Hz, 2”- and 6”-H), 7.79 (s, 1H, 5’-H); IR (neat, cm^−1^) 3477, 2949, 1460, 1215, 1070, 1047, 818, 559. ESI-MS: 504 (M+H)^+^; Anal. Calcd for C_32_H_45_N_3_O_2_: C, 76.30; H, 9.00; N, 8.34. Found: C, 76.17; H, 8.82; N, 8.56.

*16β-[4-(4-Methoxyphenyl)-1H-1,2,3-triazol-1-ylmethyl]androst-5-ene-3β,17β-diol* (**7e**): Eluent: CH_2_Cl_2_/EtOAc (70:30), yielding **7e **as a white solid (205 mg, 86%), mp 262-264 °C, *R*_f_ = 0.39 (ss C); ^1^H-NMR (DMSO-*d_6_*); δ [ppm] = 0.76 (s, 3H, 18-CH_3_), 0.86-0.90 (m, 2H), 0.95 (s, 3H, 19-CH_3_), 1.03-1.18 (overlapping m, 3H), 1.33-1.43 (overlapping m, 4H), 1.52 (m, 2H), 1.66 (m, 1H), 1.75-1.87 (overlapping m, 3H), 2.08-2.14 (m, 2H), 2.64 (m, 1H, 16-H), 3.24 (m, 1H, 3-H), 3.71 (dd, 1H, *J* = 9.5 Hz, *J* = 3.0 Hz, 17-H), 3.79 (s, 3-H, 4”-OCH_3_), 4.17 (m, 1H, 16a-H), 4.58 (overlapping m, 2H, 3-OH and 16a-H), 4.98 (d, 1H, *J* = 3.0 Hz, 17-OH), 5.21 (br s, 1H, 6-H), 7.00 (d, 2H, *J* = 8.5 Hz, 3”- and 5”-H), 7.75 (d, 2H, *J* = 8.5 Hz, 2”- and 6”-H), 8.44 (s, 1H, 5’-H); IR (neat, cm^−1^) 3454, 3206, 2930, 1499, 1250, 1068, 1028, 833, 667. ESI-MS: 478 (M+H)^+^; Anal. Calcd for C_29_H_39_N_3_O_3_: C, 72.92; H, 8.23; N, 8.80. Found: C, 73.11; H, 8.05; N, 8.97

*16β-[4-(2-Methoxyphenyl)-1H-1,2,3-triazol-1-ylmethyl]androst-5-ene-3β,17β-diol* (**7f**): Eluent: CH_2_Cl_2_/EtOAc (70:30), yielding **7f **as a white solid (208 mg, 87%), mp 219-220 °C, *R*_f_ = 0.46 (ss C); ^1^H-NMR (DMSO-*d_6_*); δ [ppm] = 0.76 (s, 3H, 18-CH_3_), 0.86 (m, 2H), 0.94 (s, 3H, 19-CH_3_), 1.02-1.15 (overlapping m, 3H), 1.32-1.41 (overlapping m, 4H), 1.50 (m, 2H), 1.66 (m, 1H), 1.74-1.85 (overlapping m, 3H), 2.09-2.13 (m, 2H), 2.65 (m, 1H, 16-H), 3.24 (m, 1H, 3-H), 3.70 (dd, 1H, *J* = 9.5 Hz, *J* = 3.0 Hz, 17-H), 3.90 (s, 3H, 2”-OCH_3_), 4.22 (m, 1H, 16a-H), 4.59 (overlapping m, 2H, 3-OH and 16a-H), 4.97 (d, 1H, *J* = 3.0 Hz, 17-OH), 5.20 (br s, 1H, 6-H), 7.03 (t, 1H, *J* = 7.0 Hz, 5”-H), 7.10 (d, 1H, *J* = 8.0 Hz, 3”-H), 7.31 (t, 1H, *J* = 7.0 Hz, 4”-H), 8.13 (d, 1H, *J* = 7.5 Hz, 6”-H), 8.39 (s, 1H, 5’-H); IR (neat, cm^−1^) 3408, 3252, 2941, 1489, 1246, 1045, 1020, 752. ESI-MS: 478 (M+H)^+^; Anal. Calcd for C_29_H_39_N_3_O_3_: C, 72.92; H, 8.23; N, 8.80. Found: C, 73.10; H, 8.39; N, 8.59.

*16β-[4-(4-Fluorophenyl)-1H-1,2,3-triazol-1-ylmethyl]androst-5-ene-3β,17β-diol* (**7g**): Eluent: CH_2_Cl_2_/ EtOAc (70:30), yielding **7g **as a white solid (212 mg, 91%), mp 282-283 °C, *R*_f_ = 0.43 (ss C); ^1^H-NMR (DMSO-*d_6_*); δ [ppm] = 0.76 (s, 3H, 18-CH_3_), 0.87 (m, 2H), 0.95 (s, 3H, 19-CH_3_), 1.04-1.14 (overlapping m, 3H), 1.30-1.43 (overlapping m, 4H), 1.53 (m, 2H), 1.64 (m, 1H), 1.75-1.87 (overlapping m, 3H), 2.07-2.14 (m, 2H), 2.63 (m, 1H, 16-H), 3.24 (m, 1H, 3-H), 3.71 (d, 1H, *J* = 9.5 Hz, 17-H), 4.18 (m, 1H, 16a-H), 4.60 (m, 1H, 16a-H), 5.22 (br s, 1H, 6-H), 7.27 (t, 2H, *J* = 8.0 Hz, 3”- and 5”-H), 7.86 (t, 2H, *J* = 8.0 Hz, 2”- and 6”-H), 8.57 (s, 1H, 5’-H); IR (neat, cm^−1^) 3426, 2941, 1558, 1495, 1231, 1051, 817, 607. ESI-MS: 466 (M+H)^+^; Anal. Calcd for C_28_H_36_FN_3_O_2_: C, 72.23; H, 7.79; N, 9.02. Found: C, 72.44; H, 7.66; N, 8.84.

*16β-[4-(3-Aminophenyl)-1H-1,2,3-triazol-1-ylmethyl]androst-5-ene-3β,17β-diol* (**7h**): Eluent: CH_2_Cl_2_/ EtOAc (50:50), yielding **7h **as a white solid (190 mg, 82%), mp 227-228 °C, *R*_f_ = 0.22 (ss C); ^1^H-NMR (DMSO-*d_6_*); δ [ppm] = 0.76 (s, 3H, 18-CH_3_), 0.89 (m, 2H), 0.95 (s, 3H, 19-CH_3_), 1.05-1.13 (overlapping m, 3H), 1.34-1.52 (overlapping m, 6H), 1.66 (m, 1H), 1.76-1.89 (overlapping m, 3H), 2.07-2.12 (m, 2H), 2.63 (m, 1H, 16-H), 3.24 (m, 1H, 3-H), 3.70 (dd, 1H, *J* = 9.5 Hz, *J* = 3.0 Hz, 17-H), 4.18 (m, 1H, 16a-H), 4.57 (overlapping m, 2H, 3-OH and 16a-H), 4.98 (d, 1H, *J* = 3.0 Hz, 17-OH), 5.13 (br s, 2H, NH_2_), 5.21 (br s, 1H, 6-H), 6.51 (d, 1H, *J* = 7.0 Hz, 4”-H), 6.92 (d, 1H, *J* = 7.0 Hz, 6”-H), 7.05 (t, 1H, *J* = 7.0 Hz, 5”-H), 7.08 (s, 1H, 2”-H), 8.39 (s, 1H, 5’-H); IR (neat, cm^−1^) 3558, 3373, 2936, 1585, 1439, 1066, 1046, 790, 586. ESI-MS: 463 (M+H)^+^; Anal. Calcd for C_28_H_38_N_4_O_2_: C, 72.69; H, 8.28; N, 12.11. Found: C, 72.86; H, 8.45; N, 12.39.

*16β-[4-(2-Pyridyl)-1H-1,2,3-triazol-1-ylmethyl]androst-5-ene-3β,17β-diol* (**7i**): Eluent: CH_2_Cl_2_/ EtOAc (50:50), yielding **7i **as a white solid (202 mg, 90%), mp 240-241 °C, *R*_f_ = 0.26 (ss C); ^1^H-NMR (10% MeOD/CDCl_3_); δ [ppm] = 0.76 (s, 3H, 18-CH_3_), 0.85 (m, 2H), 0.95 (s, 3H, 19-CH_3_), 1.04-1.14 (overlapping m, 3H), 1.37-1.53 (overlapping m, 6H), 1.60 (m, 1H), 1.79-1.91 (overlapping m, 3H), 2.13-2.20 (m, 2H), 2.64 (m, 1H, 16-H), 3.41 (m, 1H, 3-H), 3.76 (d, 1H, *J* = 10.0 Hz, 17-H), 4.27 (dd, 1H, *J* = 13.5 Hz, *J* = 8.0 Hz, 16a-H), 4.67 (dd, 1H, *J* = 13.5 Hz, *J* = 6.0 Hz, 16a-H), 5.24 (br s, 1H, 6-H), 7.21 (m, 1H, 4”-H), 7.76 (t, 1H, *J* = 7.5 Hz, 5”-H), 8.09 (d, 1H, *J* = 7.5 Hz, 6”-H), 8.22 (s, 1H, 5’-H), 8.46 (d, 1H, *J* = 3.0 Hz, 3”-H); IR (neat, cm^−1^) 3331, 2929, 1599, 1460, 1263, 1072, 787, 577. ESI-MS: 449 (M+H)^+^; Anal. Calcd for C_27_H_36_N_4_O_2_: C, 72.29; H, 8.09; N, 12.49. Found: C, 72.40; H, 8.22; N, 12.41.

*16β-(4-Cyclopropyl-1H-1,2,3-triazol-1-ylmethyl)androst-5-ene-3β,17β-diol* (**7j**): Eluent: CH_2_Cl_2_/ EtOAc (75:25), yielding **7j **as a white solid (170 mg, 83%), mp 235-236 °C, *R*_f_ = 0.47 (ss C); ^1^H-NMR (DMSO-*d_6_*); δ [ppm] = 0.67 (m, 2H), 0.72 (s, 3H, 18-CH_3_), 0.81-0.89 (overlapping m, 4H), 0.94 (s, 3H, 19-CH_3_), 1.02-1.13 (overlapping m, 3H), 1.31-1.53 (overlapping m, 6H), 1.66 (m, 1H), 1.77-1.91 (overlapping m, 3H), 2.08-2.14 (m, 2H), 2.57 (m, 1H, 16-H), 3.24 (m, 1H, 3-H), 3.67 (dd, 1H, *J* = 9.5 Hz, *J* = 3.5 Hz, 17-H), 4.05 (m, 1H, 16a-H), 4.46 (m, 1H, 16a-H), 4.61 (br s, 1H, 3-OH), 4.95 (d, 1H, *J* = 3.5 Hz, 17-OH), 5.23 (br s, 1H, 6-H), 7.79 (s, 1H, 5’-H); IR (neat, cm^−1^) 3401, 3251, 2937, 1433, 1219, 1049, 808, 588. ESI-MS: 412 (M+H)^+^; Anal. Calcd for C_25_H_37_N_3_O_2_: C, 72.95; H, 9.06; N, 10.21. Found: C, 73.19; H, 9.27; N, 10.36.

### 3.8. General procedure for preparation of 3β-acetoxy-16β-(5-substituted-1H-tetrazol-1-ylmethyl)-androst-5-en-17β-ols ***9a-e***

Compound **5** (387 mg, 1 mmol) was dissolved in CH_2_Cl_2_ (5 mL), and Cu_2_(OTf)_2_·C_6_H_6_ (50 mg, 10 mol %) was added as catalyst. The appropriate nitrile (1.1 mmol) was added to the reaction mixture, which was then stirred for 48 h at ambient temperature. The progress of the reactions was monitored by TLC, and the solvent was then evaporated *in vacuo.* The resulting crude product was purified by flash chromatography with CH_2_Cl_2_/EtOAc (95:5) as eluent.

*3β-Acetoxy-16β-(5-methoxycarbonyl-1H-tetrazol-1-ylmethyl)androst-5-en-17β-ol* (**9a**): Nitrile: methyl cyanoformate (**8a**, 0.09 mL) was added to the mixture. After purification, **9a** was obtained as a white solid (310 mg, 66%), mp 168-171 °C, *R*_f_ = 0.47 (ss B); ^1^H-NMR (CDCl_3_); δ [ppm] = 0.85 (s, 3H, 18-CH_3_), 0.90-0.97 (overlapping m, 2H), 1.02 (s, 3H, 19-CH_3_), 1.09-1.22 (overlapping m, 3H), 1.44-1.58 (overlapping m, 5H), 1.71 (m, 1H), 1.83-1.86 (overlapping m, 3H), 1.94 (m, 1H), 2.01 (s, 3H, Ac-CH_3_), 2.30 (m, 2H, 4-H_2_), 2.86 (m, 1H, 16-H), 3.84 (d, 1H, *J* = 9.5 Hz, 17-H), 4.05 (s, 3H, OCH_3_), 4.57 (m, 1H, 3-H), 4.66 (dd, 1H, *J* = 13.5 Hz, *J* = 8.0 Hz, 16a-H), 5.04 (dd, 1H, *J* = 13.5 Hz, *J* = 7.0 Hz, 16a-H), 5.34 (d, 1H, *J* = 4.0 Hz, 6-H); ^13^C-NMR (CDCl_3_); δ [ppm] = 11.8 (C-18), 18.9 (C-19), 20.1 (C-11), 21.0 (Ac-CH_3_), 27.3, 30.0, 30.8, 31.1, 36.2, 36.6, 36.7, 37.6, 39.8, 43.4, 49.4, 49.6, 51.0, 53.3 (OCH_3_), 73.4 (C-3), 80.5 (C-17), 121.6 (C-6), 139.4 (C-5), 145.4 (C-5’), 156.8, 170.2 (Ac-CO); IR (neat, cm^−1^) 3501, 2934, 1742, 1703, 1427, 1254, 1032, 826, 691. ESI-MS: 473 (M+H)^+^; Anal. Calcd for C_25_H_36_N_4_O_5_: C, 63.54; H, 7.68; N, 11.86. Found: C, 63.75; H, 7.57; N, 12.03.

*3β-Acetoxy-16β-(5-ethoxycarbonyl-1H-tetrazol-1-ylmethyl)androst-5-en-17β-ol* (**9b**): Nitrile: ethyl cyanoformate (**8b**, 0.11 mL) was added to the mixture. After purification, **9b** was obtained as a white solid (350 mg, 72%), mp 172-174 °C, *R*_f_ = 0.59 (ss B); ^1^H-NMR (CDCl_3_); δ [ppm] = 0.84 (s, 3H, 18-CH_3_), 0.91-0.96 (overlapping m, 2H), 1.01 (s, 3H, 19-CH_3_), 1.08-1.16 (overlapping m, 2H), 1.21 (m, 1H), 1.45 (t, 3H, *J* = 7.0 Hz, OEt-CH_3_), 1.47-1.58 (overlapping m, 5H), 1.70 (m, 1H), 1.82-1.86 (overlapping m, 3H), 1.92 (m, 1H), 2.01 (s, 3H, Ac-CH_3_), 2.30 (m, 2H, 4-H_2_), 2.85 (m, 1H, 16-H), 3.84 (d, 1H, *J* = 9.5 Hz, 17-H), 4.51 (q, 2H, *J* = 7.0 Hz, OEt-CH_2_), 4.56 (m, 1H, 3-H), 4.66 (dd, 1H, *J* = 13.5 Hz, *J* = 8.5 Hz, 16a-H), 5.03 (dd, 1H, *J* = 13.5 Hz, *J* = 7.0 Hz, 16a-H), 5.33 (d, 1H, *J* = 4.0 Hz, 6-H); ^13^C-NMR (CDCl_3_); δ [ppm] = 12.2 (C-18), 14.0, 19.3 (C-19), 20.4 (C-11), 21.4 (Ac-CH_3_), 27.6, 30.3, 31.1, 31.5, 36.6, 36.9, 37.1, 38.0, 40.2, 43.8, 49.7, 50.0, 51.3, 63.4, 73.7 (C-3), 80.8 (C-17), 121.9 (C-6), 139.7 (C-5), 145.9 (C-5’), 156.8, 170.5 (Ac-CO); IR (neat, cm^−1^) 3428, 2918, 1743, 1721, 1470, 1240, 1020, 854. ESI-MS: 487 (M+H)^+^; Anal. Calcd for C_26_H_38_N_4_O_5_: C, 64.18; H, 7.87; N, 11.51. Found: C, 64.32; H, 7.67; N, 11.66.

*3β-Acetoxy-16β-(5-benzyloxycarbonyl-1H-tetrazol-1-ylmethyl)androst-5-en-17β-ol* (**9c**): Nitrile: benzyl cyanoformate (**8c** 0.16 mL) was added to the mixture. After purification, **9c** was obtained as a white solid (340 mg, 62%), mp 153-156 °C, *R*_f_ = 0.21 (ss A); ^1^H-NMR (CDCl_3_); δ [ppm] = 0.78 (s, 3H, 18-CH_3_), 0.88-0.95 (overlapping m, 2H), 1.02 (s, 3H, 19-CH_3_), 1.08-1.15 (overlapping m, 3H), 1.43-1.57 (overlapping m, 5H), 1.66 (m, 1H), 1.78-1.86 (overlapping m, 3H), 1.96 (m, 1H), 2.01 (s, 3H, Ac-CH_3_), 2.31 (m, 2H, 4-H_2_), 2.79 (m, 1H, 16-H), 3.76 (d, 1H, *J* = 9.5 Hz, 17-H), 4.59-4.63 (overlapping m, 2H, 3- and 16a-H), 5.00 (dd, 1H, *J* = 13.5 Hz, *J* = 7.0 Hz, 16a-H), 5.33 (d, 1H, *J* = 3.0 Hz, 6-H), 5.46 (dd, 2H, *J* = 21.5 Hz, *J* = 12.0 Hz, OCH_2_Ph), 7.37 (m, 3H, 3”-, 4”- and 5”-H), 7.46 (d, 2H, *J* = 7.0 Hz, 2”- and 6”-H); ^13^C-NMR (CDCl_3_); δ [ppm] = 12.1 (C-18), 19.3 (C-19), 20.4 (C-11), 21.4 (Ac-CH_3_), 27.6, 30.2, 31.1, 31.5, 36.6, 36.9, 37.1, 38.0, 40.2, 43.7, 49.7, 50.0, 51.3, 68.7, 73.7 (C-3), 80.8 (C-17), 122.0 (C-6), 128.7 (2C), 128.9 (2C), 129.0 (C-4”), 134.0 (C-1”), 139.7 (C-5), 145.9 (C-5’), 156.6, 170.5 (Ac-CO); IR (neat, cm^−1^) 3525, 2945, 1733, 1703, 1454, 1256, 1026, 748, 697. ESI-MS: 549 (M+H)^+^; Anal. Calcd for C_31_H_40_N_4_O_5_: C, 67.86; H, 7.35; N, 10.21. Found: C, 67.98; H, 7.52; N, 10.12

*3β-Acetoxy-16β-(5-acetyl-1H-tetrazol-1-ylmethyl)androst-5-en-17β-ol* (**9d**): Nitrile: acetyl cyanide (**8d**, 0.08 mL) was added to the mixture. After purification, **9d** was obtained as a white solid (260 mg, 57%), mp 191-193 °C, *R*_f_ = 0.33 (ss A); ^1^H-NMR (CDCl_3_); δ [ppm] = 0.82 (s, 3H, 18-CH_3_), 0.91-0.97 (overlapping m, 2H), 1.01 (s, 3H, 19-CH_3_), 1.09-1.19 (overlapping m, 3H), 1.44-1.59 (overlapping m, 5H), 1.72 (m, 1H), 1.81-1.87 (overlapping m, 3H), 1.95 (m, 1H), 2.01 (s, 3H, Ac-CH_3_), 2.31 (m, 2H, 4-H_2_), 2.53 (s, 3H, 5’Ac-CH_3_), 2.84 (m, 1H, 16-H), 3.81 (d, 1H, *J* = 9.5 Hz, 17-H), 4.56 (m, 1H, 3-H), 4.64 (dd, 1H, *J* = 13.5 Hz, *J* = 7.5 Hz, 16a-H), 5.02 (dd, 1H, *J* = 13.5 Hz, *J* = 7.0 Hz, 16a-H), 5.33 (d, 1H, *J* = 3.5 Hz, 6-H); ^13^C-NMR (CDCl_3_); δ [ppm] = 11.9 (C-18), 19.1 (C-19), 20.2 (C-11), 20.6, 21.0 (Ac-CH_3_), 27.4, 30.2, 30.9, 31.3, 36.4, 36.8, 37.0, 37.8, 39.9, 43.6, 49.5, 49.9, 51.2, 73.5 (C-3), 80.7 (C-17), 121.7 (C-6), 139.6 (C-5), 147.7 (C-5’), 170.4 (Ac-CO), 190.4; IR (neat, cm^−1^) 3512, 2931, 1740, 1709, 1486, 1259, 1023, 896, 682. ESI-MS: 457 (M+H)^+^; Anal. Calcd for C_25_H_36_N_4_O_4_: C, 65.76; H, 7.95; N, 12.27. Found: C, 65.61; H, 8.06; N, 12.51.

*3β-Acetoxy-16β-(5-benzoyl-1H-tetrazol-1-ylmethyl)androst-5-en-17β-ol* (**9e**): Nitrile: benzoyl cyanide (**8e**, 145 mg) was added to the mixture. After purification, **9e** was obtained as a white solid (280 mg, 54%), mp 182-185 °C, *R*_f_ = 0.30 (ss A); ^1^H-NMR (CDCl_3_); δ [ppm] = 0.81 (s, 3H, 18-CH_3_), 0.88-0.95 (overlapping m, 2H), 1.02 (s, 3H, 19-CH_3_), 1.08-1.12 (overlapping m, 2H), 1.23 (m, 1H), 1.43-1.61 (overlapping m, 5H), 1.73-1.86 (overlapping m, 4H), 1.93 (m, 1H), 2.01 (s, 3H, Ac-CH_3_), 2.30 (m, 2H, 4-H_2_), 2.88 (m, 1H, 16-H), 3.82 (d, 1H, *J* = 9.5 Hz, 17-H), 4.57 (m, 1H, 3-H), 4.64 (dd, 1H, *J* = 13.5 Hz, *J* = 7.5 Hz, 16a-H), 5.03 (dd, 1H, *J* = 13.5 Hz, *J* = 7.0 Hz, 16a-H), 5.33 (d, 1H, *J* = 4.0 Hz, 6-H), 7.54 (t, 2H, *J* = 7.5 Hz, 3”- and 5”-H), 7.69 (t, 1H, *J* = 7.5 Hz, 4”-H), 8.33 (d, 2H, *J* = 7.5 Hz, 2”- and 6”-H); ^13^C-NMR (CDCl_3_); δ [ppm] = 11.8 (C-18), 18.9 (C-19), 20.1 (C-11), 21.0 (Ac-CH_3_), 27.3, 30.0, 30.7, 31.1, 36.2, 36.6, 36.8, 37.6, 39.9, 43.4, 49.4, 49.6, 50.8, 73.4 (C-3), 80.7 (C-17), 121.6 (C-6), 128.4 (2C), 130.6 (2C), 134.5 (C-4”), 134.7 (C-1”), 139.3 (C-5), 149.5 (C-5’), 170.2 (Ac-CO), 181.8; IR (neat, cm^−1^) 3533, 2938, 1728, 1702, 1595, 1265, 1026, 921, 692. ESI-MS: 519 (M+H)^+^; Anal. Calcd for C_30_H_38_N_4_O_4_: C, 69.47; H, 7.38; N, 10.80. Found: C, 69.63; H, 7.21; N, 10.91.

### 3.9. 16β-Azidomethylandrost-5-ene-3β,17β-diol (***10***)

Compound **5** (1.94 g, 5 mmol) was dissolved in MeOH (80 mL), and KOH (750 mg, 13.5 mmol) was added. The mixture was stirred for 1 h at room temperature, and then poured into water (800 mL) and neutralized with diluted HCl. The resulting precipitate was filtered off, washed with water and dried. The crude product obtained was purified by flash chromatography to afford **10** as a white solid (1.43 g, 83%), mp 154-157 °C, *R*_f_ = 0.19 (ss A); ^1^H-NMR (CDCl_3_); δ [ppm] = 0.78 (s, 3H, 18-CH_3_), 0.93 (m, 1H), 1.02 (s, 3H, 19-CH_3_), 1.05-1.15 (overlapping m, 3H), 1.42-1.60 (overlapping m, 6H), 1.84-1.89 (overlapping m, 4H), 1.99 (m, 1H), 2.25-2.32 (m, 2H, 4-H_2_), 2.37 (m, 1H, 16-H), 3.29 (dd, 1H, *J* = 12.0 Hz, *J* = 6.5 Hz, 16a-H), 3.52 (m, 1H, 3-H), 3.57 (dd, 1H, *J* = 12.5 Hz, *J* = 7.5 Hz, 16a-H), 3.78 (dd, 1H, *J* = 9.5 Hz, *J* = 5.0 Hz, 17-H), 5.34 (d, 1H, *J* = 4.5 Hz, 6-H); ^13^C-NMR (CDCl_3_); δ [ppm] = 12.1 (C-18), 19.4 (C-19), 20.6 (C-11), 30.6, 31.2, 31.6, 31.7, 36.6, 37.2, 37.5, 39.9, 42.2, 43.6, 50.0, 50.1, 53.3, 71.6 (C-3), 81.3 (C-17), 121.2 (C-6), 140.9 (C-5); IR (neat, cm^−1^) 3519, 2941, 2904, 2115, 1452, 1374, 1246, 1028. ESI-MS: 346 (M+H)^+^; Anal. Calcd for C_20_H_31_N_3_O_2_: C, 69.53; H, 9.04; N, 12.16. Found: C, 69.38; H, 9.16; N, 12.35.

### 3.10. General procedure for preparation of 16β-(5-substituted-1H-tetrazol-1-ylmethyl)androst-5-ene-3β,17β-diols ***11a-e***

Compound **10** (345 mg, 1 mmol) was dissolved in CH_2_Cl_2_ (5 mL), and Cu_2_(OTf)_2_·C_6_H_6_ (50 mg, 10 mol %) was added as catalyst. The appropriate nitrile (1.1 mmol) was added to the reaction mixture, which was then stirred for 48 h at ambient temperature. The progress of the reactions was monitored by TLC, and the solvent was then evaporated *in vacuo.* The resulting crude product was purified by flash chromatography with CH_2_Cl_2_/EtOAc (85:15) as eluent.

*16β-(5-Methoxycarbonyl-1H-tetrazol-1-ylmethyl)androst-5-ene-3β,17β-diol* (**11a**): Nitrile: methyl cyanoformate (**8a**, 0.09 mL) was added to the mixture. After purification, **11a** was obtained as a white solid (255 mg, 59%), mp 183-185 °C, *R*_f_ = 0.19 (ss B); ^1^H-NMR (CDCl_3_); δ [ppm] = 0.86 (s, 3H, 18-CH_3_), 0.92-0.96 (overlapping m, 2H), 1.02 (s, 3H, 19-CH_3_), 1.07-1.14 (overlapping m, 3H), 1.46-1.58 (overlapping m, 5H), 1.73 (m, 1H), 1.83-1.87 (overlapping m, 3H), 1.95 (m, 1H), 2.25-2.31 (overlapping m, 2H), 2.87 (m, 1H, 16-H), 3.51 (m, 1H, 3-H), 3.85 (dd, 1H, *J* = 9.5 Hz, *J* = 3.5 Hz, 17-H), 4.06 (s, 3H, OCH_3_), 4.67 (dd, 1H, *J* = 13.5 Hz, *J* = 8.0 Hz, 16a-H), 5.04 (dd, 1H, *J* = 13.5 Hz, *J* = 7.0 Hz, 16a-H), 5.33 (d, 1H, *J* = 4.5 Hz, 6-H); ESI-MS: 431 (M+H)^+^; Anal. Calcd for C_23_H_34_N_4_O_4_: C, 64.16; H, 7.96; N, 13.01. Found: C, 64.43; H, 7.80; N, 13.19.

*16β-(5-Ethoxycarbonyl-1H-tetrazol-1-ylmethyl)androst-5-ene-3β,17β-diol* (**11b**): Nitrile: ethyl cyanoformate (**8b**, 0.11 mL) was added to the mixture. After purification, **11b** was obtained as a white solid (285 mg, 64%), mp 176-179 °C, *R*_f_ = 0.27 (ss B); ^1^H-NMR (CDCl_3_); δ [ppm] = 0.86 (s, 3H, 18-CH_3_), 0.93-0.96 (overlapping m, 2H), 1.02 (s, 3H, 19-CH_3_), 1.07-1.15 (overlapping m, 3H), 1.46 (t, 3H, *J* = 7.0 Hz, OEt-CH_3_), 1.49-1.59 (overlapping m, 5H), 1.72 (m, 1H), 1.82-1.86 (overlapping m, 3H), 1.96 (m, 1H), 2.23-2.30 (overlapping m, 2H), 2.86 (m, 1H, 16-H), 3.51 (m, 1H, 3-H), 3.85 (dd, 1H, *J* = 9.0 Hz, *J* = 3.0 Hz, 17-H), 4.53 (q, 2H, *J* = 7.0 Hz, OEt-CH_2_), 4.67 (dd, 1H, *J* = 13.5 Hz, *J* = 8.0 Hz, 16a-H), 5.04 (dd, 1H, *J* = 13.5 Hz, *J* = 7.5 Hz, 16a-H), 5.32 (d, 1H, *J* = 3.5 Hz, 6-H); ESI-MS: 445 (M+H)^+^; Anal. Calcd for C_24_H_36_N_4_O_4_: C, 64.84; H, 8.16; N, 12.60. Found: C, 64.97; H, 8.36; N, 12.84.

*16β-(5-Benzyloxycarbonyl-1H-tetrazol-1-ylmethyl)androst-5-ene-3β,17β-diol* (**11c**): Nitrile: benzyl cyanoformate (**8c**, 0.16 mL) was added to the mixture. After purification, **11c** was obtained as a white solid (268 mg, 53%), mp 178-181 °C, *R*_f_ = 0.38 (ss B); ^1^H-NMR (CDCl_3_); δ [ppm] = 0.79 (s, 3H, 18-CH_3_), 0.87-0.94 (overlapping m, 2H), 1.02 (s, 3H, 19-CH_3_), 1.05-1.15 (overlapping m, 3H), 1.42-1.55 (overlapping m, 5H), 1.67 (m, 1H), 1.78-1.85 (overlapping m, 3H), 1.91 (m, 1H), 2.24-2.32 (overlapping m, 2H), 2.80 (m, 1H, 16-H), 3.51 (m, 1H, 3-H), 3.76 (dd, 1H, *J* = 9.5 Hz, *J* = 3.0 Hz, 17-H), 4.63 (dd, 1H, *J* = 13.5 Hz, *J* = 8.0 Hz, 16a-H), 5.01 (dd, 1H, *J* = 13.5 Hz, *J* = 7.5 Hz, 16a-H), 5.32 (d, 1H, *J* = 4.0 Hz, 6-H), 5.47 (q, 2H, *J* = 12.0 Hz, OCH_2_Ph), 7.38 (m, 3H, 3”-, 4”- and 5”-H), 7.48 (d, 2H, *J* = 6.5 Hz, 2”- and 6”-H); ESI-MS: 507 (M+H)^+^; Anal. Calcd for C_29_H_38_N_4_O_4_: C, 68.75; H, 7.56; N, 11.06. Found: C, 68.88; H, 7.74; N, 10.89.

*16β-(5-Acetyl-1H-tetrazol-1-ylmethyl)androst-5-ene-3β,17β-diol* (**11d**): Nitrile: acetyl cyanide (**8d**, 0.08 mL) was added to the mixture. After purification, **11d** was obtained as a white solid (195 mg, 47%), mp 199-202 °C, *R*_f_ = 0.41 (ss B); ^1^H-NMR (CDCl_3_); δ [ppm] = 0.84 (s, 3H, 18-CH_3_), 0.93-0.97 (overlapping m, 2H), 1.02 (s, 3H, 19-CH_3_), 1.08-1.16 (overlapping m, 3H), 1.47-1.58 (overlapping m, 5H), 1.74 (m, 1H), 1.82-1.87 (overlapping m, 3H), 1.96 (m, 1H), 2.26-2.32 (overlapping m, 2H), 2.55 (s, 3H, 5’Ac-CH_3_), 2.86 (m, 1H, 16-H), 3.51 (m, 1H, 3-H), 3.83 (d, 1H, *J* = 9.5 Hz, 17-H), 4.66 (dd, 1H, *J* = 13.5 Hz, *J* = 7.5 Hz, 16a-H), 5.03 (dd, 1H, *J* = 13.5 Hz, *J* = 7.0 Hz, 16a-H), 5.32 (d, 1H, *J* = 3.5 Hz, 6-H); ESI-MS: 415 (M+H)^+^; Anal. Calcd for C_23_H_34_N_4_O_3_: C, 66.64; H, 8.27; N, 13.52. Found: C, 66.48; H, 8.38; N, 13.74.

*16β-(5-Benzoyl-1H-tetrazol-1-ylmethyl)androst-5-ene-3β,17β-diol* (**11e**): Nitrile: benzoyl cyanide (**8e**, 145 mg) was added to the mixture. After purification, **11e** was obtained as a white solid (215 mg, 45%), mp 196-200 °C, *R*_f_ = 0.34 (ss B); ^1^H-NMR (CDCl_3_); δ [ppm] = 0.83 (s, 3H, 18-CH_3_), 0.91-0.96 (overlapping m, 2H), 1.03 (s, 3H, 19-CH_3_), 1.07-1.14 (overlapping m, 3H), 1.46-1.57 (overlapping m, 5H), 1.71 (m, 1H), 1.81-1.86 (overlapping m, 3H), 1.94 (m, 1H), 2.23-2.31 (overlapping m, 2H), 2.87 (m, 1H, 16-H), 3.51 (m, 1H, 3-H), 3.84 (d, 1H, *J* = 9.5 Hz, 17-H), 4.66 (dd, 1H, *J* = 13.5 Hz, *J* = 7.5 Hz, 16a-H), 5.03 (dd, 1H, *J* = 13.5 Hz, *J* = 7.0 Hz, 16a-H), 5.32 (d, 1H, *J* = 4.0 Hz, 6-H), 7.56 (t, 2H, *J* = 7.5 Hz, 3”- and 5”-H), 7.70 (t, 1H, *J* = 7.5 Hz, 4”-H), 8.34 (d, 2H, *J* = 7.5 Hz, 2”- and 6”-H); ESI-MS: 477 (M+H)^+^; Anal. Calcd for C_28_H_36_N_4_O_3_: C, 70.56; H, 7.61; N, 11.76. Found: C, 70.77; H, 7.45; N, 11.89.

### 3.11. Determination of Antiproliferative Activities

Human cancer cell lines were purchased from ECACC (Salisbury, UK). HeLa (cervix adenocarcinoma), A2780 (ovarian carcinoma) and MCF7 (breast adenocarcinoma) cells were cultivated in minimal essential medium supplemented with 10% fetal bovine serum, 1% non-essential amino acids and an antibiotic-antimycotic mixture.

Near-confluent cancer cells were seeded onto a 96-well microplate (5000/well) and attached to the bottom of the well overnight. On the second day, new medium containing the tested compound (at 10 or 30 µM, 200 μL) was added. After incubation for 72 h at 37 °C in humidified air containing 5% CO_2_, the living cells were assayed by the addition of 5 mg/mL MTT solution (20 μL). MTT was converted by intact mitochondrial reductase and precipitated as blue crystals during a 4 h contact period. The medium was then removed and the precipitated crystals were dissolved in 100 μL DMSO during a 60 min period of shaking at 25 °C. Finally, the reduced MTT was assayed at 545 nm, using a microplate reader; wells with untreated cells were utilized as controls [31]. All *in vitro* experiments were carried out on two microplates with at least five parallel wells. Cisplatin was used as positive control. Stock solutions of the tested substances (10 mM) were prepared with DMSO. The DMSO content of the medium (0.1% or 0.3%) did not have any significant effect on the cell proliferation.

## 4. Conclusions

In summary, the efficient syntheses of several D-ring-substituted steroidal triazoles and tetrazoles were achieved by means of 1,3-dipolar cycloadditions. The simple and fast reactions were carried out under mild conditions that furnished the desired compounds in good yields. The novel synthesized compounds were screened for their activities against a panel of three human gynecological cancer cell lines (HeLa, MCF7 and A2780). The application of “click” chemistry to further sterane skeletons was encouraged by these promising results.
